# Did the COVID‐19 pandemic impact on time trends in self‐poisoning in youth? A sex‐specific national register‐based interrupted time‐series analysis in Sweden

**DOI:** 10.1111/bjc.70064

**Published:** 2026-05-05

**Authors:** Katelyn Battista, Cecilia Magnusson, Nicola Orsini, Michael Lundberg, Anna Ohlis, Gergö Hadlaczky, Lucie Laflamme

**Affiliations:** ^1^ Department of Global Public Health Karolinska Institutet Stockholm Sweden; ^2^ Department of Health Sciences Brock University St. Catharines Ontario Canada; ^3^ Center for Epidemiology and Community Medicine Region Stockholm Stockholm Sweden; ^4^ National Centre for Suicide Research and Prevention LIME, Karolinska Institutet Stockholm Sweden; ^5^ Center for Health Economics, Informatics and Health Care Research Region Stockholm Stockholm Sweden; ^6^ Institute for Social and Health Sciences University of South Africa Pretoria South Africa

**Keywords:** COVID‐19, depression, interrupted time series, mental ill‐health, poisoning, self‐harm, youth

## Abstract

**Objectives:**

To assess the extent to which the COVID‐19 pandemic impacted trends of self‐poisoning in young boys and girls in Sweden, and whether impacts differed by sociodemographic and psychiatric health factors.

**Methods:**

We used an interrupted time series analysis of a register‐based national cohort to assess the sex‐specific associations between the COVID‐19 pandemic and monthly rate of hospitalization for intentional self‐poisoning. Individual‐level data were collected from a rolling cohort of Swedish young people ages 10–19 between 2005 and 2021 (approximately 1.13 million individuals per year). Separate models were run to examine differential associations among girls by age group, socioeconomic position, and prior anxiety or depression diagnosis.

**Results:**

A total of 17,151 cases of self‐poisoning were observed across the study period, 78.4% among girls. The association between the pandemic and self‐poisoning was sex‐specific. An annualized increase of 19% per year (95% CI: 5%–35%) between March 2020 and December 2021 occurred among girls, corresponding to a reversal of a declining pre‐pandemic trend (*p* = .0002). No significant change in level or trend occurred for boys. Increases among girls were higher among ages 10–14 and were observed regardless of socioeconomic position or prior psychiatric diagnosis.

**Conclusion:**

In Sweden, despite a less restrictive public health response, the COVID‐19 pandemic was accompanied by a substantial increase in rates of self‐poisoning and a reversal of previously declining trends among young girls, exacerbating long‐standing gender gaps. This underscores the need for enhanced preparedness to deal with indirect effects of future health crises on this dangerous expression of mental distress.

**Practitioner points**

**Significant outcomes**

- In Sweden, the COVID‐19 pandemic exacerbated longstanding gender differences in self‐poisoning among young people ages 10–19 years, with young girls most negatively affected.
- The COVID‐19 pandemic led to a reversal of previously declining trends in self‐poisoning among young girls, with levels reaching or surpassing peaks last seen in 2005.
- Increases in self‐poisoning due to the COVID‐19 pandemic were widespread among girls regardless of socioeconomic position or prior psychiatric condition and were largest among girls ages 10–14 years.

**Limitations**

- Our study did not examine the substance used in poisoning due to registry data limitations.
- Our study did not examine differential associations by sociodemographic or psychiatric health factors among boys due to low case counts.
- Our study only examined cases of self‐poisoning that required hospitalization and therefore does not capture less severe occurrences such as those reported to the poison centres or treated only in the emergency department.

## INTRODUCTION

Mental ill‐health among young people is a globally recognized public health issue that can be both worsened by and deprioritized during periods of societal crisis. Syntheses of the aftermath of the COVID‐19 pandemic show it has exacerbated already preoccupying negative trends in severe conditions like depression and anxiety, particularly among young girls (Samji et al., 2022). Alongside the worsening of mental health, there is a growing body of knowledge showing that the pandemic has been associated with increases in self‐poisoning, by far the most common form of self‐harm leading to hospitalization among young people (Buckley et al., 2025; Gilley et al., 2020). Evidence from settings across the United States (Apata et al., 2023; Farah et al., 2023; McCabe et al., 2023), Australia (Buckley et al., 2025) and Europe (Koppen et al., 2023; Reneflot et al., 2023; Singh et al., 2024) shows an increase in self‐poisoning among young people attributed to the COVID‐19 pandemic. Additional clinical‐based studies in Australia (Gatenby et al., 2023), South Korea (An et al., 2024; Park et al., 2022), Pakistan (Kidwai et al., 2023) and Europe (Azkunaga et al., 2023; Predescu et al., 2023) show increases in the proportion of ED visits due to self‐poisoning during the pandemic period. Identified increases occurred across varying outcome indicators and substances, and commonly aligned with the imposition of public health‐related restrictions (Buckley et al., 2025; Gatenby et al., 2023).

Evidence on the impacts of the COVID‐19 pandemic can provide relevant insights and implications for future global health crises causing population‐level disruption. Despite being largely convergent, the increases identified thus far are highly dependent on the differing country‐specific pandemic contexts and corresponding public health responses. Additionally, the current body of knowledge on the impact of COVID‐19 suffers from a few significant shortcomings. A first is that the study designs are relatively weak: either based on small clinical samples and lacking comparator populations or significance testing, or else constructed as ‘pre‐during’ studies with short pre‐pandemic periods. A limited pre‐pandemic examination period lacks important contextualizing information on prior long‐term and seasonal trends, which in turn limits the possibility to assess potential trajectory changes or sustained impacts. A few population‐based studies have examined trends in self‐poisoning over relatively longer periods of time; however, the observation periods end prior to the pandemic. Studies from North America (Gilley et al., 2020; Spiller et al., 2019) have identified prior downward trends in incidences of self‐poisoning from the early 2000's followed by upward trends beginning in the 2010's. Similar increases beginning in 2011–2012 were observed in Australia (Cairns et al., 2019; Chitty et al., 2022). An earlier study from the United Kingdom found increases in poisonings from over the counter and prescription medications among young girls from 1998 to 2013 (Tyrrell et al., 2018). The magnitude of increase varies by study, which may reflect country‐specific pre‐pandemic contexts. Necessary contextualizing information on pre‐pandemic trends in Sweden and other parts of Europe remains lacking.

Another shortcoming of the current evidence is that insufficient attention is paid to the role of risk factors known for their association with self‐poisoning like low socioeconomic position (Pawer et al., 2021) and poor mental health, in particular depression and anxiety (Samji et al., 2022). The one exception to this is the examination of differences by sex. Studies that examined sex differences in self‐poisoning consistently found a greater impact of the pandemic on girls than boys (Buckley et al., 2025; Farah et al., 2023; Koppen et al., 2023; Predescu et al., 2023; Reneflot et al., 2023; Singh et al., 2024). Further, the only study to examine the post‐pandemic period found that the increases among girls partially persisted after the removal of restrictions (Buckley et al., 2025). However, while the incidence of self‐poisoning is consistently higher among girls than boys over time (Cairns et al., 2019; Spiller et al., 2019), the trends among girls from higher‐risk groups, such as those from socioeconomically disadvantaged families or those with pre‐existing mental health conditions, remain largely unexamined.

In light of these knowledge gaps, the primary objective of our study, set at Swedish national level, was therefore to examine sex‐specific changes in rates and trends of hospitalization for intentional self‐poisoning among Swedish young people following the onset of the COVID‐19 pandemic and associated public health restrictions in March 2020. A secondary objective was to examine potential differential associations among girls by age group, family socioeconomic position, and prior diagnosis for anxiety or depression.

## MATERIALS AND METHODS

### Design

This study uses an interrupted time series design to assess the effect of the onset of the COVID‐19 pandemic and the corresponding imposition of public health advisories and restrictions in March 2020 on long‐term trends in hospitalized cases of self‐poisoning in Sweden.

### COVID‐19 in Sweden

The World Health Organization (WHO) declared COVID‐19 a pandemic on 11 March 2020. The Swedish response to COVID‐19 is generally considered to be less restrictive than peer countries and largely limited to voluntary measures with no general lockdown (Ludvigsson, 2020). As summarized by Ludvigsson (Ludvigsson, 2020), the first case of COVID‐19 in Sweden was recognized on 31 January 2020, following which the Swedish National Board of Health classified COVID‐19 as a disease dangerous to the public, with a subsequent high alert issued following a second case on 26 February 2020. Between 10 March and 30 March, public health advisories related to unnecessary travel, physical distancing and working from home were enacted, while gatherings over 50 people and visits to elderly care homes were banned. Schools were closed for students over 17 years old, while younger students remained in in‐person learning. Given the rapid implementation of advisories and restrictions during the month of March 2020, this period was identified as an appropriate interruption point for the time series analysis.

Notably, initial travel restrictions were eased by 1 July, with in‐person learning for students over 17 years old resuming on 17 August (Ludvigsson, 2020). While additional advisories and density restrictions were enacted in subsequent waves between October 2020 and December 2021, no formal lockdowns were ever enacted.

### Data source

We use data from the Developmental Origins of Health and Disease (DOHaD) national cohort (Madley‐Dowd et al., 2022, 2024), which follows individuals born between 1973 and 2021 who resided in Sweden at some point during that period. DOHaD links register information from the Total Population Register (TPR), National Patient Register (NPR), Prescribed Drug Register (PDR), Multigeneration Register (MGR), the Cause of Death Register (CDR) and the Longitudinal Integration Database for Occupational Research (LISA).

### Participants

This study covers the period 2005–2021 and includes girls and boys who were between the ages of 10–19 and resided in Sweden at some point during that period. Individuals are included in the cohort beginning in the calendar year in which they turn 10 years old and excluded when they turn 20 years old. The approximate cohort size is 1.13 million individuals per year, with an average of around 1300 cases of hospitalization for self‐harm per year. This study focuses on incident cases of self‐poisoning; participants are removed from the analytic cohort following the first self‐poisoning occurrence.

### Measures

#### Hospitalization for intentional self‐poisoning

The outcome is hospitalization for intentional self‐poisoning, classified according to WHO's International Classification of Diseases 10th Revision (ICD‐10) as X60‐X69. Only incident cases of non‐fatal hospital treatment in inpatient or specialized outpatient care are included. Within the cohort, this corresponds to 17,151 total incident cases (13,449 for girls, 3702 for boys). Cases resulting in death with or without prior hospitalization (128 for girls, 90 for boys) are excluded. The registry data does not include cases of poisoning that did not necessitate hospital treatment, for instance those treated in primary care or calls to poison information centres.

#### Sociodemographic factors

Individual participant sex (female, male) and age group (10–14 years, 15–19 years) are determined from the NPR. Additional family characteristics are determined by linking to parent information from the MGR, NPR and LISA. Factors related to family socioeconomic position include the number of parents born in Sweden (none, one, both), family income quintile at the time of the participant's birth, and mother's education level at the time of the participant's birth (primary, secondary, post‐secondary).

#### Prior anxiety or depression diagnosis

Prior diagnosis of anxiety is determined from the NPR based on ICD‐10 codes F40‐F43, while prior diagnosis of depression is determined based on ICD‐10 codes F32‐F39. Only diagnoses made within inpatient or specialized outpatient care are included since the NPR does not include data on diagnoses made in primary care. The reference period for a ‘prior’ diagnosis is any date before the date of incident self‐poisoning occurrence.

### Statistical analysis

We used Quasi‐Poisson regression models to examine the change in trends in monthly rates of hospitalization for intentional self‐poisoning before and after March 2020. The regression models included 0th‐ and 1st‐degree calendar time splines (months) with a knot in March 2020: the former captures a level shift, the latter a change in linear trend. Separate models were estimated for girls and boys. We adjusted for seasonal variation using Fourier terms, with the number of sine‐cosine pairs chosen through comparison of model BICs: three pairs were selected for girls and two pairs for boys. Quasi‐Poisson models include a scaling term to account for overdispersion. Further details of this ITS analysis approach are available (Bernal et al., 2017).

Next, we examined whether the onset of the COVID‐19 pandemic was associated with differential changes in self‐poisoning by sociodemographic or psychiatric health factors using interaction terms between calendar time and each covariate—age group, parents born in Sweden, family income group, mother's education level, prior anxiety diagnosis and prior depression diagnosis—in separate regression models. Evidence for interaction was assessed using Wald tests at the 5% significance level. Because monthly case counts for boys were small, these interaction analyses were conducted only for girls.

Lastly, we conducted sensitivity testing to examine potential sex‐specific non‐linear pre‐pandemic trends. We used an exploratory change point analysis to identify additional trend inflection points prior to March 2020 and recalculated the corresponding level shifts. (See Supporting Information for additional methodological details and results).

R software version 4.4.3 was used for all analyses. The R packages ‘tsModel’ and ‘glm’ were used to construct and test ITS models, and the package ‘ggplot’ was used for plotting.

### Ethics

The register data used for this project have been gathered in the context of a research project that has received approval by the Swedish Ethical Review Authority, with the following ethical approval numbers: dnr: 2020‐05516; 2020‐05516; 2021‐05958‐02; 2022‐05648‐02, with co‐author CM as PI. The ethical approvals cover the studies proposed herein.

## RESULTS

### Cohort characteristics

Table 1 shows the average population size and total number of cases of hospitalization for self‐poisoning over the study period 2005–2021 for boys and girls in each sociodemographic and psychiatric diagnosis group. There were 17,151 cases of hospitalization for self‐poisoning across the study period, 78.4% of which occurred among girls (*n* = 13,449). Across both sexes, the proportion of cases was substantially higher among ages 15–19 years, those in the bottom 60% family income bracket, and those whose mother's education level at birth was primary or secondary. While only 2.5% and 1.9% of the cohort had prior anxiety or depression diagnoses, respectively, nearly one‐third of all cases occurred in these groups.

**TABLE 1:** Sex‐stratified cohort characteristics and total cases of hospitalization for intentional self‐poisoning throughout the study period.

	Girls				Boys			
	Average cohort size 2005–2021		Total cases 2005–2021		Average cohort size 2005–2021		Total cases 2005–2021	
	*n*	%	*n*	%	*n*	%	*n*	%
Total	546,246	100.0	13,449	100.0	582,447	100.0	3702	100.0
**Age group**								
10–14 years	267,513	49.0	2911	21.6	282,693	48.5	401	10.8
15–19 years	278,733	51.0	10,538	78.4	299,754	51.5	3301	89.2
**Parents Born in Sweden**								
None	86,864	15.9	1812	13.5	93,478	16.0	391	10.6
One or Both	431,819	79.1	10,581	78.7	457,640	78.6	2897	78.3
[Missing]	27,563	5.0	1056	7.9	31,328	5.4	414	11.2
**Family income group**								
Top 40%	308,738	56.5	3866	28.7	327,037	56.1	1024	27.7
Bottom 60%	162,941	29.8	7554	56.2	172,605	29.6	2056	55.5
[Missing]	74,567	13.7	2029	15.1	82,805	14.2	622	16.8
**Mother's education level**								
Primary or Secondary	201,882	37.0	8841	65.7	213,519	36.7	2435	65.8
Post‐Secondary	270,572	49.5	2558	19.0	286,949	49.3	635	17.2
[Missing]	73,792	13.5	2050	15.2	81,979	14.1	632	17.1
**Prior anxiety diagnosis**								
No	528,911	96.8	8743	65.0	571,541	98.1	2752	74.3
Yes	17,335	3.2	4706	35.0	10,906	1.9	950	25.7
**Prior depression diagnosis**								
No	535,267	98.0	9203	68.4	535,267	91.9	2843	76.8
Yes	10,979	2.0	4246	31.6	10,979	1.9	859	23.2

### Sex‐specific trends in hospitalized self‐poisoning before and during COVID‐19

Trends in rates of hospitalization for intentional self‐poisoning from interrupted time series models are presented in Figure 1 with corresponding estimated rate ratios in Table 2. Among girls, the rate of hospitalization for self‐poisoning declined by 2.5% per year between January 2005 and March 2020 (RR = .98; 95% CI: .97–.98), after accounting for a strong seasonal variation corresponding to lower rates in summer months (June–July). An immediate 13% higher rate of hospitalization was estimated in March 2020 (RR = 1.13; 95% CI: .98–1.30), followed by a reversal in trend (*p* = .002). Between March 2020 and December 2021, every 12 months increase conferred a 19.0% higher rate of hospitalization for self‐poisoning of (RR = 1.19; 95% CI: 1.05–1.35). In absolute terms, this corresponds to an annualized increase of 64.2 cases per 100,000 between February 2020 and December 2021.

**FIGURE 1:**
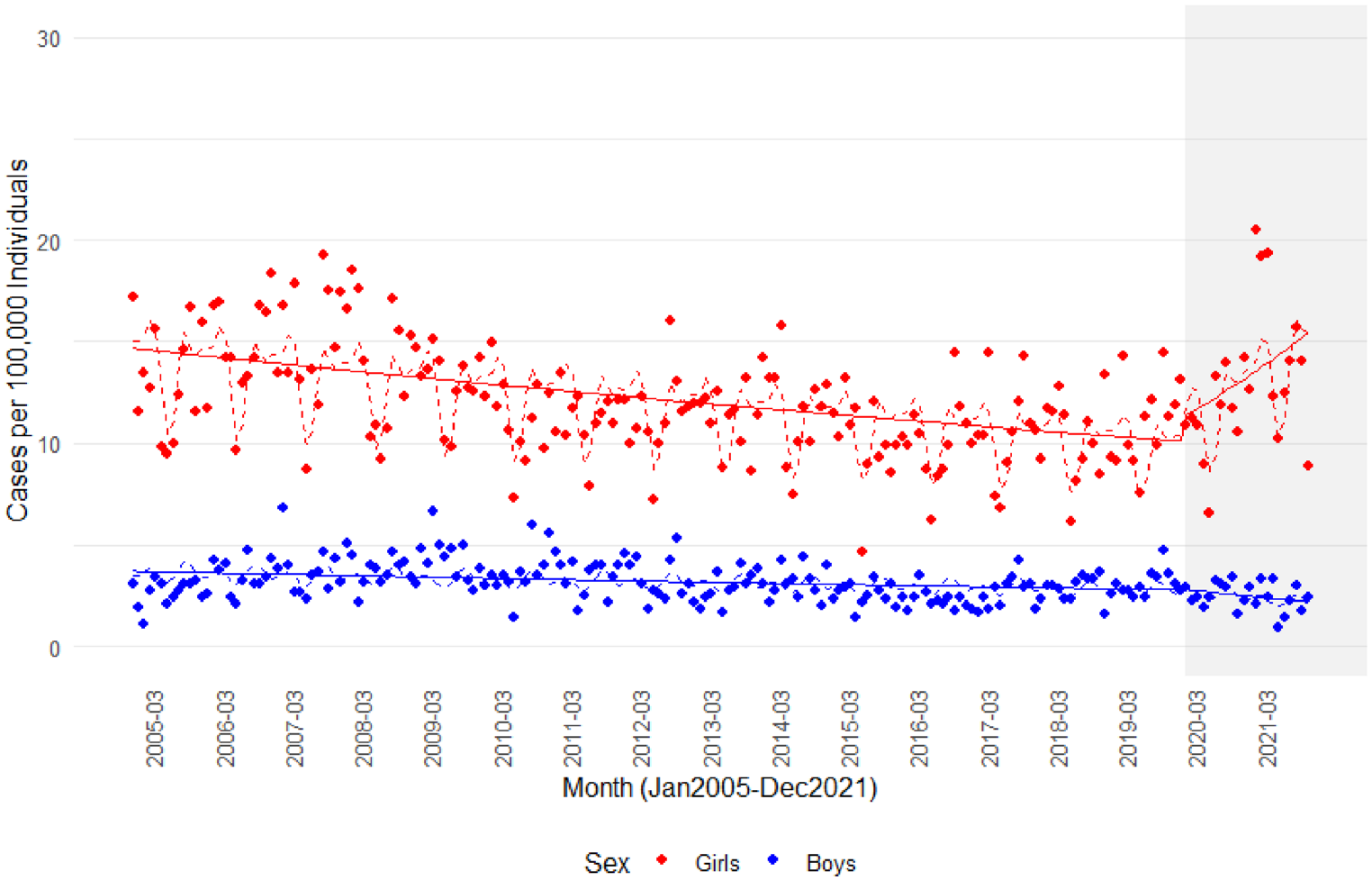
Sex‐stratified monthly rates of hospitalization for intentional self‐poisoning (cases per 100,000 individuals). Fitted interrupted time series models for level and slope change at March 2020 are presented with seasonality adjustment (dotted line) and deseasonalized trend (solid line).

**TABLE 2:** Estimated rate ratios for annual linear trends before and after March 2020 and level change at March 2020 from fitted interrupted time series models among boys and girls ages 10–19 in Sweden.

		Annual trend January 2005 to March 2020 (RR per year)		Level change at March 2020		Annual trend March 2020 to December 2021 (RR per year)	
		RR	95% CI	RR	95% CI	RR	95% CI
**Overall**							
Boys		.98	(.97, .99)	1.01	(.78, 1.30)	.87	(.68, 1.11)
Girls		.98	(.97, .98)	1.13	(.98, 1.30)	1.19	(1.05, 1.35)
**Girls: by sociodemographic group**							
Age group (years)	10–14	.98	(.97, .99)	1.44	(1.13, 1.84)	1.34	(1.09, 1.64)
	15–19	.98	(.97, .99)	1.04	(.89, 1.22)	1.12	(.98, 1.29)
Parents born in Sweden	None	.96	(.94, .97)	1.17	(.86, 1.6)	1.20	(.92, 1.58)
	One or Both	.98	(.97, .98)	1.14	(.98, 1.31)	1.21	(1.07, 1.37)
Family income group	Top 40%	.96	(.95, .97)	1.27	(1, 1.6)	1.12	(.91, 1.38)
	Bottom 60%	.98	(.98, .99)	1.08	(.92, 1.27)	1.25	(1.08, 1.44)
Mother's education level	Primary or Secondary	.99	(.98, .99)	.99	(.84, 1.18)	1.28	(1.11, 1.49)
	Post‐Secondary	.97	(.96, .98)	1.58	(1.25, 1.99)	1.11	(.91, 1.35)
Prior anxiety diagnosis	No	.94	(.94, .95)	1.12	(.93, 1.34)	1.24	(1.06, 1.45)
	Yes	.93	(.92, .94)	1.20	(1, 1.44)	1.06	(.9, 1.24)
Prior depression diagnosis	No	.96	(.95, .96)	1.15	(.97, 1.35)	1.16	(1, 1.34)
	Yes	.94	(.93, .94)	1.19	(.97, 1.44)	1.16	(.98, 1.38)

Abbreviation: RR = rate ratio.

Among boys, a similar decreasing trend in rates of hospitalization for self‐poisoning of 1.9% per year (RR = .98; 95% CI: .97–.99) was seen between January 2005 and March 2020, though at a lower magnitude and with less seasonal variation than seen for girls. No substantial change in level (RR = 1.01; 95% CI: .78–1.30) or trend (*p* = .332) was observed after March 2020 (RR = .87; 95% CI: .68–1.11).

Sensitivity testing for non‐linear pre‐pandemic trends revealed two additional inflection points among girls, corresponding to a gradual levelling of rates beginning in May 2008 (RR = .99; 95% CI: .98–1.00) followed by modest but non‐significant increase in rates beginning in June 2015 (RR = 1.02; 95% CI: .99–1.05) (see Figure S1 and Table S2). These pre‐pandemic trend adjustments largely accounted for the level change seen in March 2020 (revised RR = 1.01; 95% CI: .87–1.17); however, the increasing trend following March 2020 remained significantly higher than the pre‐pandemic period (*p* = .002). No meaningful pre‐pandemic trend changes were identified for boys (see Table S1).

### Differential changes among girls by sociodemographic factors

Trends in rates of hospitalization for intentional self‐poisoning among girls are compared across sociodemographic factors in Figure 2, with corresponding estimated rate ratios in Table 2. An increase in hospitalization rates following March 2020 is evident across all sociodemographic groups (*p* < .001 for all estimated combined level and slope interactions).

**FIGURE 2:**
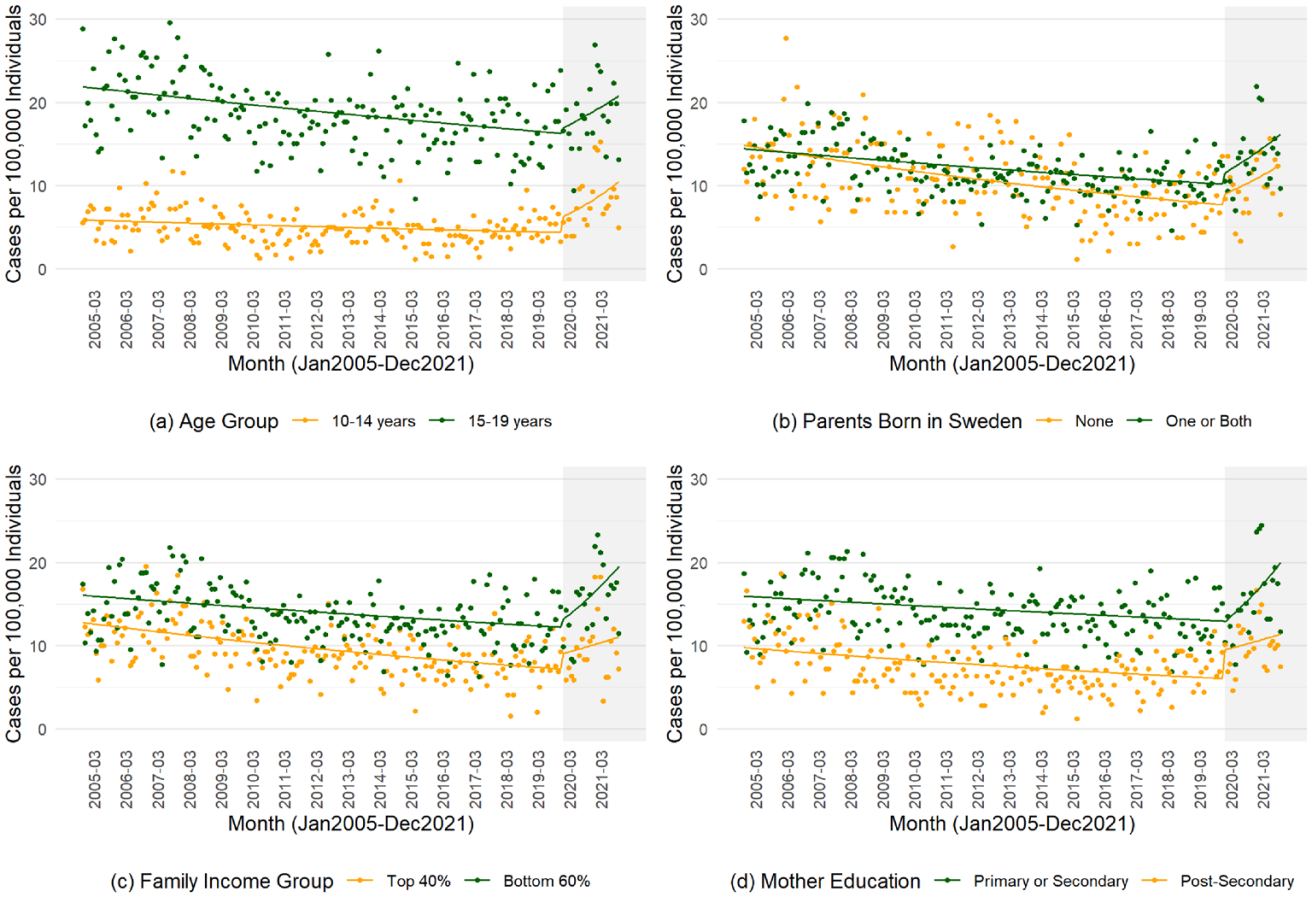
Monthly rate of hospitalization for intentional self‐poisoning per 100,000 population and fitted interrupted time series model for level and slope change in March 2020 with deseasonalized trend (solid line) among girls by: (a) age group, (b) parents born in Sweden, (c) family income group at child's birth and (d) mother's education level at child's birth.

A higher immediate increase in rates of hospitalization for self‐poisoning was seen among girls ages 10–14 years compared to those ages 15–19 years (RR: 1.38, 95% CI: 1.03–1.84). While the subsequent increasing linear trend was statistically similar for both age groups (*p* = .163), the overall increase in rates by December 2021 was 1.88 times higher for girls ages 10–14 years (95% CI: 1.43–2.47) compared to those ages 15–19 years. In absolute terms, this corresponds to an annualized increase of 73.2 cases per 100,000 among girls 10–14 and 54.0 cases per 100,000 among girls 15–19 between February 2020 and December 2021.

While pre‐COVID trends differed, no significant differences in overall trend changes following COVID‐19 onset were seen for those whose parents were or were not born in Sweden or for those in lower vs. higher income groups. A higher immediate increase in rates of hospitalization for self‐poisoning was seen among girls whose mother had a post‐secondary education level (RR: 1.59, 95% CI: 1.19–2.11) compared to those with only primary or secondary education levels; however, this was offset by a shallower subsequent increasing trend, resulting in similar overall increases between groups by December 2021 (RR: 1.26, 95% CI: .96–1.67).

### Differential changes among girls by prior anxiety and depression diagnoses

Trends in rates of hospitalization for intentional self‐poisoning among girls are compared for those with and without prior anxiety or depression diagnoses in Figure 3, with corresponding rate ratios in Table 2. An increase in rates following COVID‐19 onset in March 2020 was evident across all groups (*p* < .001 for all estimated combined level and slope interactions). While the magnitude of hospitalization rates is substantially higher for those with a prior anxiety or depression diagnosis compared to those without, there were no significant differences in trend changes following COVID‐19 between groups. Overall proportional increases by December 2021 were similar for those with prior anxiety diagnosis compared to those without (relative rate ratio .83, 95% CI: .65–1.05) and for those with prior depression diagnosis compared to those without (relative rate ratio 1.08, 95% CI: .84–1.38). In absolute terms, this corresponds to annualized increases between February 2020 and December 2021 of 137.7 cases per 100,000 among those with anxiety diagnosis and 32.6 among those without. Corresponding increases were 561.7 and 24.7 cases per 100,000 among those with and without depression diagnosis.

**FIGURE 3:**
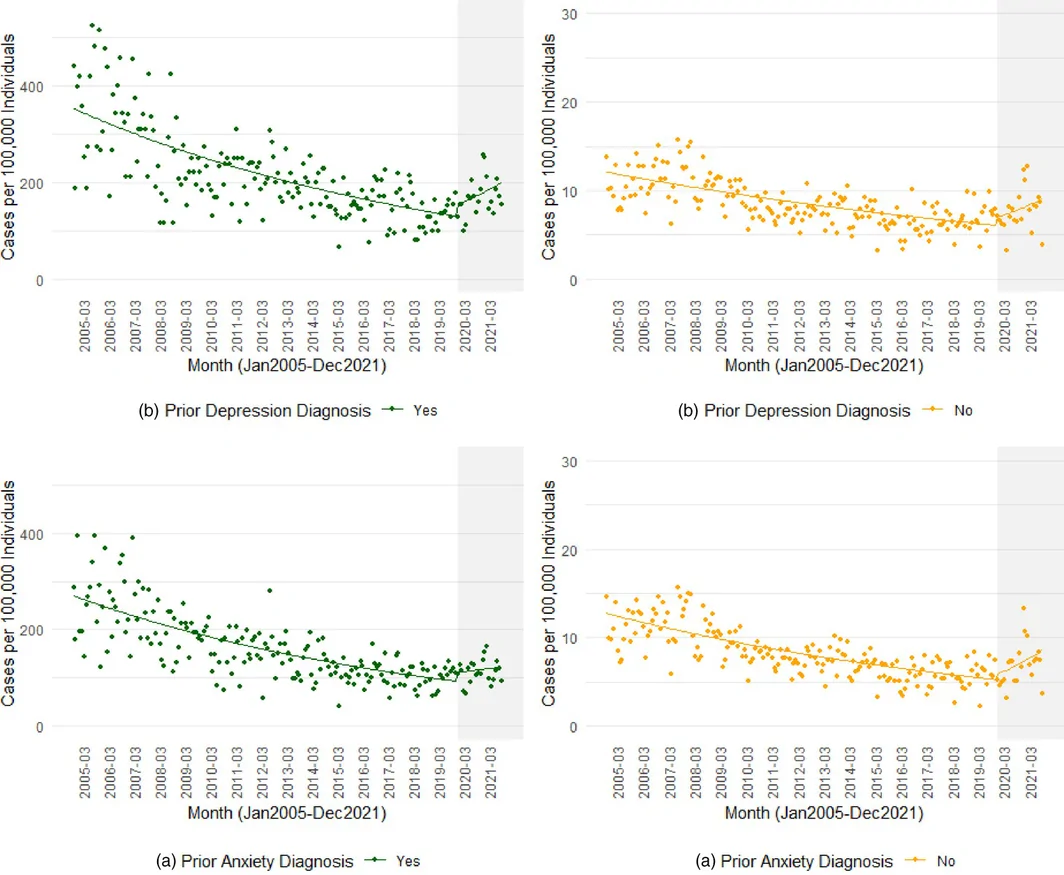
Monthly rate of hospitalization for intentional self‐poisoning per 100,000 population and fitted interrupted time series model for level and slope change in March 2020 with deseasonalized trend (solid line) among girls by (a) prior anxiety diagnosis, and (b) prior depression diagnosis. Note the difference in *y*‐axis scale for those with and without prior diagnoses.

## DISCUSSION

In Sweden as in many other settings, intentional self‐poisoning in youth is not only the most prevalent form of self‐harm leading to hospitalization (Buckley et al., 2025; Gilley et al., 2020) but also a very gendered form, as reflected by the large differences in prevalence between boys and girls in our long‐term trend data. Our results reflect a gendered influence of the pandemic on these trends, in that an immediate and sustained increase in self‐poisoning occurred among girls that was not paralleled among boys, for whom the levels remained considerably lower. For girls, the pandemic was associated with a pivotal inflection point from previous trends, resulting in self‐poisoning rates reaching or surpassing peak levels last observed in 2005. Among girls, there are important additional findings. First, changes in trend following the pandemic onset manifested differently by age group, with a most remarkable increase among girls ages 10–14, whose rates have been lower than among their older peers throughout the observation period. Second, these changes were relatively similar by family sociodemographic characteristics, considering either whether parents were born in Sweden or not, family income bracket, or maternal education level. Third, these changes were found not only among those with prior depression or anxiety diagnosis, which could be expected, but also among those without such diagnoses. Our findings suggest that the COVID‐19 pandemic has exacerbated longstanding gender differences in self‐poisoning, with a particular detrimental impact to younger girls that was widespread regardless of socioeconomic position or prior psychiatric condition.

The elevated risk of self‐poisoning among young girls is well‐established and is reflective of a broader global gender gap in adolescent mental health (Campbell et al., 2021). Our findings, which align with other studies showing greater increases in self‐poisoning due to COVID‐19 among young girls (Buckley et al., 2025; Farah et al., 2023; Koppen et al., 2023; Predescu et al., 2023; Reneflot et al., 2023), therefore highlight a pre‐existing vulnerability which the pandemic onset has exacerbated, most likely owing to the effect that it has had on the mental health of girls (Samji et al., 2022). Further, while the incidence of self‐poisoning across the study period is consistently higher among girls ages 15–19 compared to younger girls ages 10–14, we identified a significantly greater increase following the pandemic onset among the younger age group, in line with findings from Buckley et al., (2025); Reneflot et al., (2023). The increased risk to younger girls ages 10–14 caused by COVID‐19 implies a declining average initiation age, which is particularly concerning given that earlier initiation of self‐poisoning has been linked to higher rates of repetition and risk of future suicide (Finkelstein et al., 2015).

One notable distinction between our study and those from other countries is the different public health response to COVID‐19 taken by Sweden, which is viewed as far less restrictive than peer countries (Ludvigsson, 2020). Efforts to contain the pandemic in Sweden rested on recommendations rather than enforced regulations, on grounds that Swedish citizens are likely to adhere to those recommendations. Some studies from other contexts have hypothesized that the public health restrictions such as lockdowns put in place to contain the pandemic drove increases in self‐poisoning through factors such as increased isolation, lower social supports and disruptions to daily routines (Buckley et al., 2025; Gatenby et al., 2023; Koppen et al., 2023; Park et al., 2022; Reneflot et al., 2023). However, we find similar negative effects on self‐poisoning even in a context without such formal restrictions, as well as a continued increasing trend in Sweden even after many recommendations were relaxed in August 2020. This suggests that broader socioecological factors may contribute to the worsening of mental health and the pathway to self‐poisoning over this period, rather than this being a direct and acute consequence of lockdown.

One aspect of the Swedish pandemic response particularly important to this age group is that schools were kept open for those under 17 years old (Ludvigsson, 2020). Given the multifaceted negative and aggravating effects of school closure on children's mental health and wellbeing documented in several reviews (Mazrekaj & De Witte, 2024; Rajmil et al., 2021; Singh et al., 2020; Viner et al., 2022) the greater rise in rates of self‐poisoning in the 10–14 age group is therefore surprising. Indeed, a young person's increased inclination for self‐poisoning during the period may be influenced not only by their individual circumstances but also by that of their family, where all members could be affected, their peer group context, where relations could be maintained through social media (James et al., 2023), and the society as a whole, where the pandemic occupied so much space within mass media (Bao et al., 2020; Giri & Maurya, 2021). In particular, given their developmental period, younger girls in the 10–14 age group may have been more susceptible to problematic relational dynamics among family members in the home environment that exposed children to toxic stress (Rajmil et al., 2021). This age group may also have poorer emotional regulation capability leading to unhealthy and harmful coping mechanisms (Zimmermann & Iwanski, 2014). Additionally, the likelihood of a generational effect among younger girls that translates into progressively worsening mental health cannot be ruled out (Borg et al., 2025; Rausch, 2023). For instance, younger generations face increased risk through exposure to social media content that promotes differing forms of self‐harm (Biernesser et al., 2020). It is crucial for further research to better understand the mechanisms triggering these increases in self‐poisoning during COVID‐19, as well as to monitor post‐pandemic trends, in order to prevent and mitigate similar patterns during future large‐scale societal disruptions.

Within the long‐term context, we identified overall declining rates of self‐poisoning among girls since 2005, with indications of levelling or modest increase since 2015. When interruptions to long‐term trends are considered, the onset of the pandemic in March 2020 therefore represents an important inflection point in self‐poisoning trends for young girls in Sweden. Our findings within Sweden notably contrast against prior trend studies from North America (Gilley et al., 2020; Spiller et al., 2019) and Australia (Cairns et al., 2019; Chitty et al., 2022), which have seen sharp increases in self‐poisoning among girls over the prior decade. Despite this different pre‐pandemic context, our findings of an immediate and continued increase in hospitalizations between March 2020 and December 2021 align with increases seen in other countries (Kidwai et al., 2023; McCabe et al., 2023; Reneflot et al., 2023). We were not able to examine whether this trend reversal was sustained in the post‐pandemic period; however, reports from the Swedish Poison Information Center (Giftinformationscentralens, 2024) suggest that intentional poisoning has continued to increase in 2022 and 2023. The one peer‐reviewed study to examine the post‐pandemic period within Australia (Buckley et al., 2025) noted a subsequent decline in self‐poisoning following December 2021 but not a full return to pre‐pandemic levels. Given the unique pre‐pandemic context within Sweden, further evidence is needed to assess the potential sustained effects of COVID‐19 on self‐poisoning behaviours in this cohort of young girls.

Low socioeconomic position is a known risk factor for self‐poisoning (Pawer et al., 2021), as is evident in the long‐term patterns across our study period. We did not identify any differential trend changes following COVID‐19 onset on self‐poisoning across measures of socioeconomic position within the Swedish context. There is a lack of comparative evidence available from other countries specific to self‐poisoning; however, global reviews of mental health effects more broadly suggest that the pandemic exacerbated socioeconomic disparities and led to worsening mental health, particularly among adolescents from socioeconomically disadvantaged families (Kim et al., 2024; Wolf & Schmitz, 2024). Notably, many of the suggested mechanisms for this differential impact on mental health, such as reduced access to school resources (Kim et al., 2024), were likely lessened by the Swedish COVID‐19 response that prioritized keeping children in schools (Ludvigsson, 2020). Thus, our findings should not imply that the pandemic equally affected the living or family conditions of girls from different socioeconomic positions but rather show that the impact of the pandemic on self‐poisoning—as a manifestation of poor mental health—cut across socioeconomic groups.

As expected, prior diagnosis with anxiety or depression was the largest risk factor for self‐poisoning among girls across the entire study period, with incidences of hospitalization 10–20 times higher than girls without prior diagnosis. The increases in hospitalized self‐poisoning resulting from COVID‐19 in this risk group therefore contribute substantially to overall increases at the population level. The increases among this group are unsurprising, given it is well established that COVID‐19 has exacerbated mental distress among those with pre‐existing mental disorders (Carvalho et al., 2022). However, we also found similar increases in self‐poisoning among girls without any prior diagnosis. This could suggest that the pandemic has triggered new manifestations of mental distress among those who were otherwise not considered to be high‐risk. This may also be reflective of worsened detection mechanisms for those in need of help within school or healthcare systems, resulting from resources being shifted as a byproduct of the pandemic response. Indeed, evidence reviews highlight a need for future action plans to maintain appropriate mental health support among children and adolescents during such times of crisis, including digital access to mental health professionals (Singh et al., 2020).

Our study is the first to quantify associations between the COVID‐19 pandemic and hospitalizations for self‐poisoning in Sweden. Our use of a robust interrupted time series design which accounts for seasonality, monthly specificity and long‐term trends contributes to the global evidence base and provides important contextualization of COVID‐19 impacts absent from prior studies. Further, our use of individual‐level data at a national scale provides new evidence on the impacts of COVID‐19 among young girls across socioeconomic and psychiatric risk factors. A key limitation of our study was that we were unable to determine the substance used in the poisoning due to limitations within the registry data, which could be of practical importance for preventive efforts. Additionally, we were unable to examine differences across sociodemographic and psychiatric risk factors among boys due to the low number of cases. Our study only examined non‐fatal cases of self‐poisoning that required inpatient or specialized outpatient care and therefore does not capture less severe occurrences such as those reported to the poison centres or treated only in primary care, nor those poisonings that resulted in death with or without prior hospital care. We may therefore be underestimating the overall impact of COVID‐19 on self‐poisoning occurrence, particularly given that concerns around disease transmission and hospital capacity may have deterred some people from seeking hospital care. Additionally, while fatal cases represent less than 1% of total cases during the 16‐year study period, we cannot assess whether this proportion of cases remained consistent across the study period or increased during the pandemic, nor whether these cases may pertain to young people with distinct risk profiles. Additionally, our measure of prior psychiatric diagnosis does not include diagnoses made in primary care, and therefore we are likely underestimating the scope of mental illness among young girls. Lastly, our study does not capture the post‐COVID period beyond December 2021 and so we are unable to determine whether the identified changes across sociodemographic and psychiatric health groups are sustained.

## CONCLUSION

In Sweden, despite a comparatively less restrictive public health response, the COVID‐19 pandemic exacerbated longstanding gender differences in self‐poisoning in young people 10–19 years and interrupted long‐term trends in girls, with a remarkable increase among the youngest ones. The impact cut across socioeconomic groups and manifested among those with and without prior psychiatric conditions. Considering the risk of repetition and future suicide associated with self‐poisoning, following up the trends and understanding the mechanisms at stake is imperative. There is an acute need for enhanced systems‐level preparedness, including improvements in early identification and support, to prevent further increases in self‐poisoning and other harmful expressions of mental distress among young girls during future global health crises.

## AUTHOR CONTRIBUTIONS

**Katelyn Battista:** Methodology; visualization; writing – original draft; conceptualization; formal analysis; writing – review and editing. **Cecilia Magnusson:** Conceptualization; writing – review and editing; project administration; investigation. **Nicola Orsini:** Writing – review and editing; methodology. **Michael Lundberg:** Data curation; writing – review and editing; methodology. **Anna Ohlis:** Writing – review and editing. **Gergö Hadlaczky:** Writing – review and editing. **Lucie Laflamme:** Project administration; supervision; conceptualization; writing – original draft; writing – review and editing; investigation.

## FUNDING INFORMATION

Preliminary work to support this manuscript was made possible thanks to initial funding from Folkhälsomyndigheten (contract awarded to LL).

## CONFLICT OF INTEREST STATEMENT

The authors have no conflicts of interest to declare.

## ETHICS STATEMENT

The register data used for this project have been gathered in the context of a research project that has received approval by the Swedish Ethical Review Authority, with the following ethics approval numbers: dnr: 2020‐05516; 2020‐05516; 2021‐05958‐02; 2022‐05648‐02, with co‐author CM as PI. The ethics approvals cover the studies proposed herein.

## Supporting information


**Table S1.** Sex‐stratified model fit comparison for interrupted time series models with sex‐specific pre‐pandemic trend changes based on exploratory change point analysis.
**Table S2.** Estimated rate ratios for segmented annual linear trends and level change at March 2020 from fitted interrupted time series models among girls (A) assuming a linear pre‐pandemic trend, and (B) assuming pre‐pandemic inflection points at May 2008 and June 2015.
**Figure S1.** Monthly rates of hospitalization for intentional self‐poisoning among girls (cases per 100,000 individuals). Fitted interrupted time series models (A) assuming a linear pre‐pandemic trend (in red), and (B) assuming pre‐pandemic inflection points at May 2008 and June 2015 (in green).

## Data Availability

Research data are not shared.
