# Supplementary material for: Did the COVID‐19 pandemic impact on time trends in self‐poisoning in youth? A sex‐specific national register‐based interrupted time‐series analysis in Sweden
Source: Br J Clin Psychol. 2026 May 5;65(4):1200–15. doi: 10.1111/bjc.70064 (PMC13655979; doi:10.1111/bjc.70064)
Supplement: Supplementary file 1 [file BJC-65-1200-s001.docx]

**Did the COVID-19 pandemic impact on time trends in self-poisoning in youth? A sex-specific national register-based interrupted time-series analysis in Sweden**

**Supplemental Methods: Sensitivity Test for Non-linear Pre-pandemic Trend**

We first conducted an exploratory change point analysis using the strucchange() package in R version 4.4.3 to identify pre-pandemic trend inflection points, separately for girls and boys. The case counts outcome was log-transformed to account for non-normality. Breakpoints were identified at monthly inflection points while restricting a minimum segment length of six months. Two pre-pandemic inflection points were identified for girls at May 2008 and June 2015, and two for boys at October 2009 and July 2017.

We then conducted a sensitivity test for non-linear pre-pandemic trends by running the sex-stratified ITS models with additional interruption points at May 2008 and June 2015 for girls and at October 2009 and July 2017 for boys. We assessed the relative model fits against the baseline scenario of a linear pre-pandemic trend by calculating Akaike’s Information Criterion (AIC) on the corresponding Poisson models for each scenario (since the allowance for additional dispersion utilizing a quasi-Poisson distribution must be removed in order to calculate AIC) (Supplemental Table 1). For boys, the revised model resulted in an AIC increase and thus was rejected in favor of the original model.

Lastly, for girls, we calculated new annualized trends and a new level impact at March 2020 accounting for the revised trend changes at each pre-pandemic inflection point, and compared these against the estimates from the original model with linear pre-pandemic trend (Supplemental Table 2, Supplemental Figure 1).

**Supplemental Results: Sensitivity Test for Non-linear Pre-pandemic Trend**

Supplemental Table 1. Sex-stratified model fit comparison for interrupted time series models with sex-specific pre-pandemic trend changes based on exploratory change point analysis

| **ITS Model with Poisson Distribution:** | **Akaike’s Information Criterion (AIC)*** | |
| --- | --- | --- |
|  | **Girls** | **Boys** |
| Original Model: Linear pre-pandemic trend | 1584.2 | 1247.7 |
| Modified Model: Two pre-pandemic interruption points  Girls: May 2008 and June 2015  Boys: October 2009 and July 2017 | 1563.3 | 1251.1 |

**lower AIC indicates better model fit; ΔAIC >10 indicates meaningfully improved model fit*

Supplemental Table 2. Estimated rate ratios for segmented annual linear trends and level change at March 2020 from fitted interrupted time series models among girls (A) assuming a linear pre-pandemic trend, and (B) assuming pre-pandemic inflection points at May 2008 and June 2015

| **Girls** | **Rate Ratio** | **95% CI** |
| --- | --- | --- |
| **(A) Linear pre-pandemic trend** |  |  |
| Annual trend January 2005 to March 2020 | 0.98 | (0.97,0.98) |
| Level change at March 2020 | 1.13 | (0.98,1.3) |
| Annual trend March 2020 to December 2021 | 1.19 | (1.05,1.35) |
| **(B) Pre-pandemic inflection points at May 2008 and June 2015** |  |  |
| Annual trend January 2005 to May 2008 | 0.98 | (0.97,0.99) |
| Annual trend May 2008 to June 2015 | 0.99 | (0.98,1.00) |
| Annual trend June 2015 to March 2020 | 1.02 | (0.99,1.05) |
| Level change at March 2020 | 1.01 | (0.87,1.17) |
| Annual trend March 2020 to December 2021 | 1.19 | (1.06,1.34) |

Supplemental Figure 1. Monthly rates of hospitalization for intentional self-poisoning among girls (cases per 100,000 individuals). Fitted interrupted time series models (A) assuming a linear pre-pandemic trend (in red), and (B) assuming pre-pandemic inflection points at May 2008 and June 2015 (in green)
